# BAuth-ZKP—A Blockchain-Based Multi-Factor Authentication Mechanism for Securing Smart Cities

**DOI:** 10.3390/s23052757

**Published:** 2023-03-02

**Authors:** Md. Onais Ahmad, Gautami Tripathi, Farheen Siddiqui, Mohammad Afshar Alam, Mohd Abdul Ahad, Mohd Majid Akhtar, Gabriella Casalino

**Affiliations:** 1 Department of Computer Science and Engineering, Jamia Hamdard, New Delhi 110062, India; 2 Department of Computer Engineering, Jamia Millia Islamia, New Delhi 110025, India; 3 Department of Computer Science, University of Bari Aldo Moro, 70125 Bari, Italy

**Keywords:** MFA, OTP, 2FA, ZKP, BAuth-ZKP, blockchain, smart cities

## Abstract

The overwhelming popularity of technology-based solutions and innovations to address day-to-day processes has significantly contributed to the emergence of smart cities. where millions of interconnected devices and sensors generate and share huge volumes of data. The easy and high availability of rich personal and public data generated in these digitalized and automated ecosystems renders smart cities vulnerable to intrinsic and extrinsic security breaches. Today, with fast-developing technologies, the classical username and password approaches are no longer adequate to secure valuable data and information from cyberattacks. Multi-factor authentication (MFA) can provide an effective solution to minimize the security challenges associated with legacy single-factor authentication systems (both online and offline). This paper identifies and discusses the role and need of MFA for securing the smart city ecosystem. The paper begins by describing the notion of smart cities and the associated security threats and privacy issues. The paper further provides a detailed description of how MFA can be used for securing various smart city entities and services. A new concept of blockchain-based multi-factor authentication named “BAuth-ZKP” for securing smart city transactions is presented in the paper. The concept focuses on developing smart contracts between the participating entities within the smart city and performing the transactions with zero knowledge proof (ZKP)-based authentication in a secure and privacy-preserved manner. Finally, the future prospects, developments, and scope of using MFA in smart city ecosystem are discussed.

## 1. Introduction

The smart city ecosystem is dependent on the underlying network infrastructure for the majority of its processes [1,2,3]. Every request and service within the ecosystem can be thought of as a kind of transaction that is performed between the user and the system (C2M/M2C) or between devices (M2M) [4,5,6]. A secure and privacy-preserving mechanism is required to restrict the illegitimate access and usage of the system and its valuable data sources. The classical “password-only” approaches for ensuring security and privacy are no longer sufficient for the current technological era [7,8]. A typical smart city consists of thousands of IoT devices and sensors, which generate and transfer a huge volume of data. These data also include personal information of the users and thus must be protected against any unauthorized access and usage. It must also be ensured that the data are generated and transferred only by legitimate devices and users. Device and user authentication mechanisms such as OAuth, Tokens, etc., provide a decent solution to this problem. The emergence of One-Time Password (OTP) has also restricted online fraud during transactional processing to a fair extent [7,8]. However, with hackers becoming progressively smarter in coming up with new techniques to overcome the OTP mechanism and fraudulently access a legitimate system with ill intentions, the currently available security mechanisms are not able to effectively counter banking and other transactional frauds.

Furthermore, there are fair chances that the SIM card may be cloned and that the hackers may access the OTP to make a fraudulent transaction on behalf of the user by impersonating them. A smart city infrastructure is based on a large number of sensor devices, along with a large number of users with unique identities. These sensor devices and users constitute the Internet of Things and Internet of Identities, respectively. Ideally, only a legitimate user can access the connected devices to perform different activities or request services. However, this is not the case in general. There are a large number of users who are always on the lookout for any security loopholes to enter IoT systems illegitimately. The recent COVID-19 pandemic has swiftly changed the ways we do business [9,10,11]. Due to the COVID-19 pandemic, the world today is witnessing a paradigm shift in various business processes. With almost all things available online, online transactions have become a preferred and convenient way of doing business. E-commerce and online transactions have brought significant convenience to consumers, manufacturers, and enterprises by ensuring better quality, increasing sales, price comparisons, improving after-sales services, saving consumers’ valuable time, promoting job creation, and so on [12,13]. However, a large portion of society is still reluctant to use online transactions in view of the fraud associated with them. The faith in technology has been further shaken by rising reports of online fraud, phishing attacks, and cybercrimes [14]. The sharp increase and sophistication in cybercrime have rendered the existing “username and password” mechanism incompetent for safeguarding the private data of users. To overcome this, OTPs and “single sign-on systems” have emerged as a preferred means to counter such frauds/cybercrime [15,16,17]. OTP is a type of two-factor authentication mechanism. In simple terms, an OTP is a time-based access token provided by the bank or application that contains numeric or alpha numeric values. There are multiple ways of delivering OTPs to users such as “soft tokens through mobile apps”, “hard tokens” (such as key fobs), and “on-demand OTPs through SMS or emails” [18]. Before the completion of the transaction, the user is prompted to enter the OTP received on the registered mobile number or the registered email id or both. Once the correct OTP is entered by the user, the transaction is completed. Even with the OTP mechanism, the possibility of SIM cloning still exists to access the OTP and fraudulently transact on the user’s behalf [19]. Another scenario could be the loss of the user’s phone, resulting in the user failing to receive the OTP and hence being incapable of completing the transaction [20]. A smart city ecosystem is a data-driven concept where all the decisions are facilitated with the help of extensive data analysis and insights extracted from the massive data generated through a large number of sensors and other intelligent devices [21,22,23]. In a smart city setup, where most of the tasks are automated, the services requested by the users and the ones delivered by the service providers are all facilitated on a network. These requests and services are executed as a transaction between the users, smart devices, and service providers.

Therefore, a smart city setup can be thought of as a larger landscape for frequent online transactions. In order to protect users, data, devices, and the information generated throughout the transactional process, novel mechanisms need to be adopted that can ensure complete privacy and security of all the entities within the smart city setup. In this paper, the role of MFA in protecting different entities of the smart city ecosystem is discussed. The paper further proposes a blockchain based MFA approach called BAuth-ZKP for providing privacy-preserved and secured means of performing transactions.

### 1.1. Key Contributions

To the best of our knowledge, this paper presents a first-of-its-kind survey covering all aspects of MFA, including the history and background of authentication mechanisms, the present-day requirements and need for authentication, and the underlying issues and challenges associated with providing an extra layer of security in the smart city context. To begin with, the manuscript helps the reader to understand the smart city ecosystem and its underlying components, along with the associated privacy and security issues and challenges. The major contributions of the paper are as follows:

- **Identification of security flaws and limitations in existing smart city setups**

An exhaustive discussion of the privacy and security issues and challenges associated with smart city ecosystems has been performed covering technical, economic, and social domains. Several security breach incidents are also discussed, which formed the basis of motivation for the present work.

- **Identification and discussion of state-of-the-art literature on the existing mechanisms for the mitigation of security and privacy issues in smart city setups.**

A detailed search was conducted to identify the state of the art of smart city privacy and security mitigation measures covering almost all verticals.

- **Identification and discussion on the need and role of MFA in smart cities.**

With the advancement of technology, the classical password-only techniques for authentication are no longer sufficient to identify the legitimacy of the users. The need and role of MFA to provide an extra layer of security for smart city are indeed highly crucial as the cases of cybercrime have been on the rise in recent years. A comprehensive discussion on the above subject is provided, including a discussion of prominent security breach cases in the recent past and the mitigation mechanisms for the same.

- **Proposed a novel smart contract-based zero knowledge proof MFA named “BAuth-ZKP” for providing a secure and privacy-preserved authentication mechanism.**

A novel smart contract-based MFA called “BAuth-ZKP” is proposed for providing authentication of the user without exposing their PII (Personal Identifiable Information). A proof of concept (PoC) for the authentication process is also provided. The unique characteristics of the blockchain make it an appropriate technology for securing various smart city services. A detailed description of blockchain for securing the smart city services is provided. Furthermore, various blockchain-based MFA approaches are also discussed in detail.

### 1.2. Paper Organization

The paper is divided into six sections. Section 2 discusses the notion of the smart city and provides a detailed description of the major security issues in a smart city ecosystem. In Section 3, the concept of the MFA mechanism is introduced in detail. The section also provides insights into the historical background and research in the area of MFA. Section 4 discusses the need for MFA in smart city ecosystems. It further highlights the role of MFA in safeguarding individual entities of the smart city ecosystem. Section 5 provides a detailed description of the proposed “BAuth-ZKP” approach for providing privacy-preserved and secured MFA mechanisms based on the concept of smart contracts. The section further discusses other blockchain-based MFA approaches. Finally, Section 6 provides the conclusion of the manuscript and discusses the future trends in MFA.

## 2. Smart Cities and Underlying Security Issues

**Figure 1:**
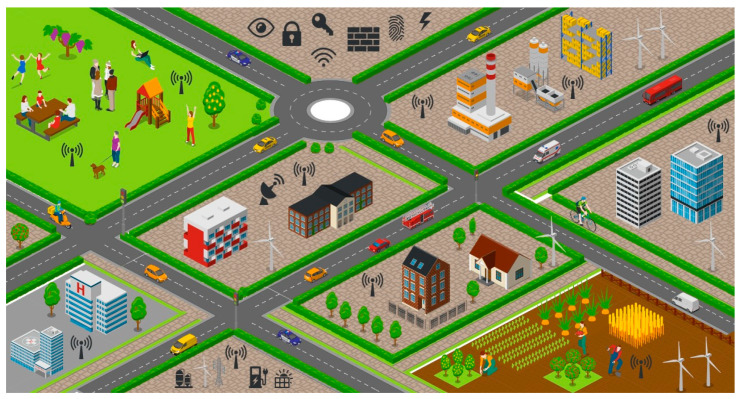
Smart city ecosystem.

Although there is no single definition of a smart city, a typical smart city can be defined as a city in which the services (healthcare, transportation, agriculture, education, constructions, industrialization, etc.) policies, governance, infrastructure, and other activities are largely automated [24,25]. Figure 1 shows a typical smart city ecosystem. As per McKinsey [26], cities that use digital solutions can improve the QoL (quality of life) of inhabitants by up to 30% as compared to inhabitants living in traditional cities. A smart city can be thought of as a relative term whose definition varies from city to city and nation to nation. There must be some qualitative and quantitative metrics to define the level of smartness of a city. These metrics depend upon several factors, including literacy rate, population, gross domestic product (GDP), economic and financial stability, personal well-being, etc. There are several intricate inherent and external issues and challenges associated with the adoption of a smart city ecosystem [27,28,29].

The privacy and security of the users and data are considered the most vital. Most of the functions of the smart city are based on automated IF-Then-Else logic. Here, the conditions are preset and embedded into the smart objects, and based on those conditions, the objects can take autonomous decisions without the need for any external interference. This reduces human efforts to a large extent and improves user convenience.

**Figure 2:**
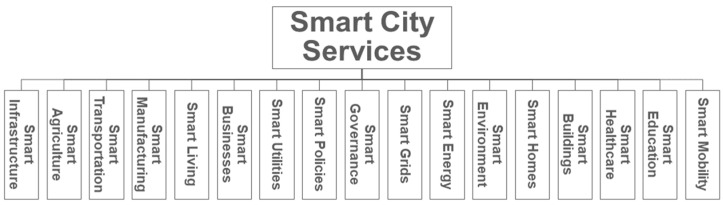
Smart city services.

Secondly, since there is limited human interference, it reduces the chances of intentional and unintentional human error(s). Smart cities provide a number of exemplary services to their inhabitants to provide better wellbeing and work–life balance with a special focus on the environment and sustainability. Figure 2 shows the services that form part of a typical smart city.

### Security Issues in a Smart City

**Figure 3:**
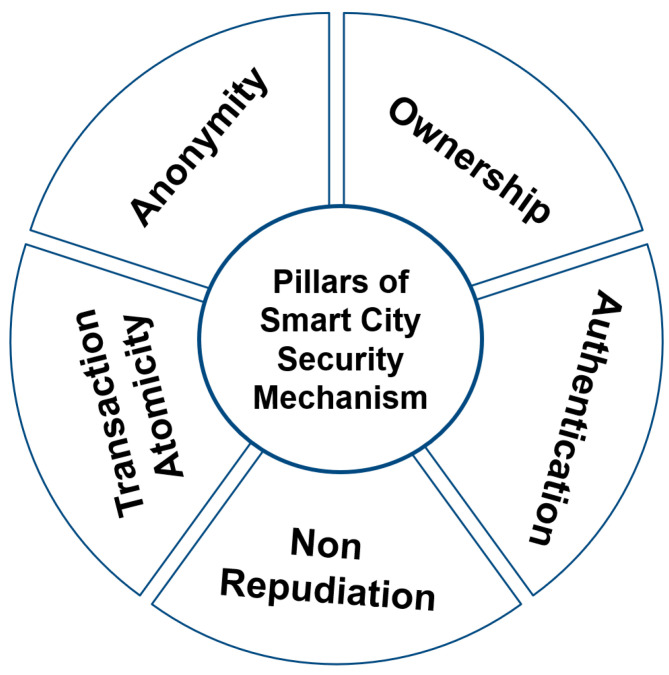
Pillars of smart city security mechanisms.

Smart city ecosystems are always prone to internal and external security threats. Since the whole smart city is dependent upon the underlying network infrastructure and the interconnections of various devices in the smart city ecosystem, the security of these networks and the participating devices is the most critical issue to be taken care of. Since most of the smart city ecosystem is built upon IoT technology, the security issues of IoT become the ultimate security issues of the smart city as well [30,31,32]. The heterogenous interconnection of devices within the smart city ecosystem makes it prone to several security threats. Additionally, there are several limitations in the available IoT devices, as most of these devices are proprietary devices that do not support interoperability. Additionally, thus, there exists a dependency issue. Figure 3 presents the pillars of smart city security.

Along with all the unprecedented benefits and applications of IoT, there are several security vulnerabilities and threats associated with it. The risk of intentionally or accidentally exposing the personal and critical information of the users, devices, and other entities of the IoT ecosystem is of prime concern, as this can result in serious consequences. Table 1 provides a summary of important discussions of smart city securities.

**Figure 4:**
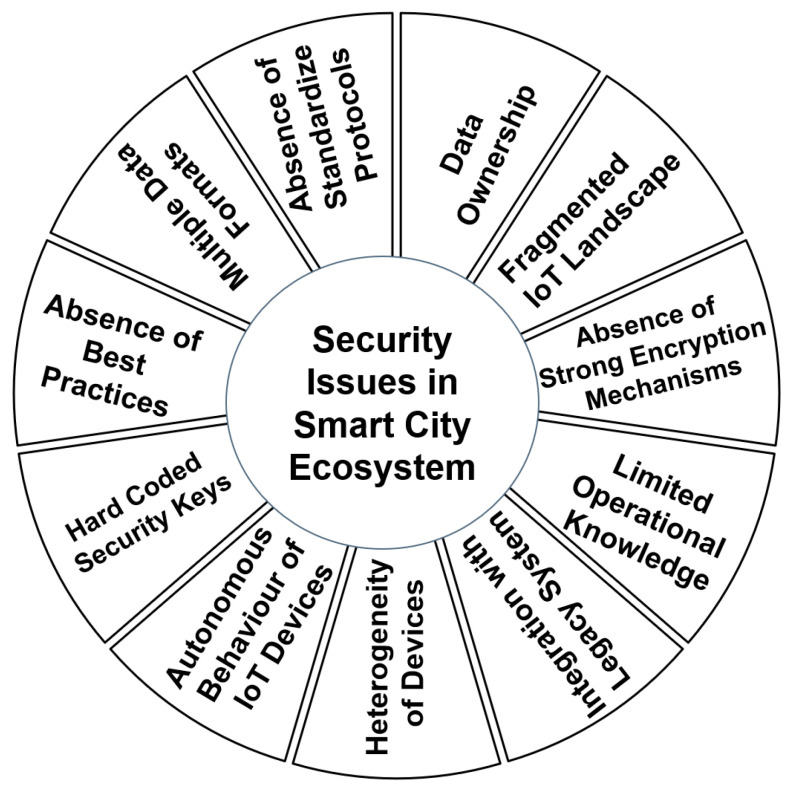
Security issues in smart city ecosystem.

Due to the lack of standard security protocols and operation manuals, IoT devices are prone to several implementation and functional risks and security issues. This is primarily because of the reasons shown in Figure 4 [30,40,51,52,53,54,55].

(1) *Heterogeneity of the Smart IoT based devices*

There are several types of IoT devices available on the market. Most of these devices are proprietary and follow their own standards and data formats, which makes it very difficult to integrate them with other IoT devices of different manufacturers. There is an urgent need to provide an interface to integrate two or more heterogenous IoT devices to make them work in sync with each other.

(2) *Varied Data collection and Storage Standards*

The different IoT devices follow their respective data collection and storage formats. All of these have different headers and data packet formats. Additionally, the data storage mechanism of these devices differs to a large extent.

(3) *Nonavailability of standard transmission protocols*

The absence of universally accepted and standard transmission protocols of IoT devices makes them vulnerable to several security threats such as DoS, DDoS, and man-in-middle attacks.

(4) *Nonavailability of universally acceptable power and space-constrained encryption standards.*

IoT devices are power-constrained devices. They have very limited computational and energy-storage capacities, and thus we cannot execute heavy encryption programs on these devices, which makes them susceptible to hacking.

(5) *Lack of standard operation manuals featuring proper use cases and misuse cases*

There are no standard operation manuals available describing the use cases and misuse cases of IoT devices within smart city ecosystems. Therefore, the end user may fall prey to the hackers without even knowing it.

(6) *Lack of standard best practices mechanism*

The responsibility of publishing the best practices lies with the manufacturer and the regulatory authorities that must publish the general and device-specific best practices of using smart devices from time to time.

(7) *Nonavailability of reachable legal avenues in the case of mishappenings*

At present, very few venues where mishappenings occurred due to IoT-enabled devices have been reported. No standard legal framework exists that can provide holistic know-how of what is good and what is bad while manufacturing, selling, purchasing, operating, and disposing of the IoT-enabled devices.

(8) *Hardcoded security keys and encryptions mechanisms*

Due to computational and space limitations, most manufacturers hard-code the security keys into the device itself. Additionally, the manufacturer usually provides the same security keys to all the devices manufactured by them, irrespective of their usage and functionalities. This makes the device more vulnerable in cases of theft or hacking when hackers gain access to the device and can alter the hardware security and encryption keys.

(9) *Data ownership issues while the data are in transit*

This is a very important aspect of security in the context of IoT devices in the smart city ecosystem. Questions such as “Who owns what data at what time and for which purpose?” are still not answered by device manufacturers and regulators. Thus, in the case of a mishappening due to the IoT device, it is unclear who will be held responsible (the device user, manufacturer, regulatory body, device seller, etc.) and to what extent.

(10) *Autonomous Behavior of IoT devices*

Most IoT devices are designed to work autonomously without depending on any external entities. On one hand, this can be thought of as an advantage; however, it has some severe vulnerabilities as well. The autonomous nature of miniature IoT devices makes them vulnerable to hacking attacks because of the limited in-built security features in the IoT devices.

(11) *Fragmented IoT landscape*

The whole IoT landscape is completely fragmented, and there is hardly any interoperability and/or general standards facilitating secure and synchronized data sharing among IoT devices of different makes and models. Thus, the IoT ecosystem currently is largely manufacturer-dependent.

(12) *Integration with legacy systems*

Legacy systems are usually built to perform high-end tasks with heavy security mechanisms inbuilt to secure such systems. Secondly, these legacy systems usually work for a specific purpose. When we try to integrate IoT devices with such systems, there are several inherent issues and challenges, which include:

    ◦ Power consumption and dissipation mechanisms
    ◦ Scalability
    ◦ Interoperability
    ◦ Standard operating procedures.

An IoT ecosystem is one where all entities that can collect and exchange data are internet-enabled, resulting in the automation of processes. With the expansion of this technology, billions of devices have been connected to the network, ranging from temperature controllers to high-end personal assistants. All these devices inherently exhibit heterogeneous hardware and software configurations and security constraints. This has resulted in a growing need for laws, regulations, and security measures governing the manufacturing, usage, and monitoring of these connected IoT devices.

## 3. Multi-Factor Authentication (MFA)

**Figure 5:**
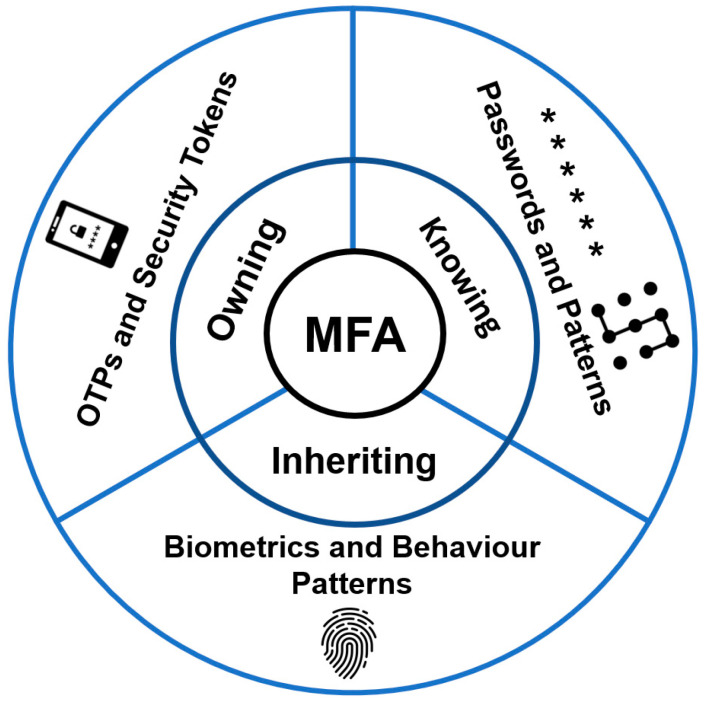
MFA criteria categories.

In the context of a smart city, every request, service, and information exchange can be thought of as a transaction where one party initiates the transaction and the other party completes the transaction. It is pertinent to ensure the legitimacy and security of these transactions. In the recent past, several mechanisms have been devised to protect transactions from illegitimate access and theft. One of the most common mechanisms is the use of multi-factor authentication. A typical multi-factor authentication mechanism is one in which the user needs to authenticate himself/herself using more than one credential. Figure 5 shows the criteria for MFA.

A one-time password (OTP) is one of the most widely used multi-factor authentication mechanisms; it requires the user to provide a numeric or alphanumeric passcode to prove their legitimacy. This OTP provides an extra layer of security over the conventional login id and password-based authentications. The main advantage of OTP is that it is valid for a single continuous session. As soon as the session is terminated, the OTP becomes invalid. There are multiple ways in which an OTP can be generated. Typically, there are two types of OTPs: time-based OTPs and event-based OTPs. Event-based OTPs are generated on the occurrence of any specific event (such as a button press or a mouse click) and are valid till the event is completed, while time-based OTPs are valid only for a specific amount of time.

**Figure 6:**
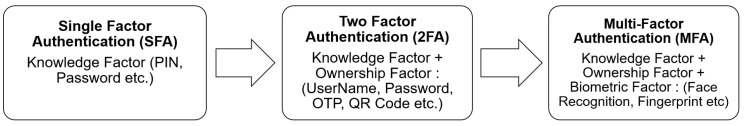
Evolution of MFA.

MFA has emerged as a security structure that necessitates multiple authentication mechanisms to validate the legitimacy of a user. MFA incorporates two or more autonomous credentials (such as password, security token, and biometric verification) to build a layered security framework or protect a target (e.g., database, computing device, network, etc.) from unauthorized access [56,57]. This section highlights the evolution from single-factor authentication (SFA) and two-factor authentication (2FA) towards MFA, as shown in Figure 6.

**Figure 7:**
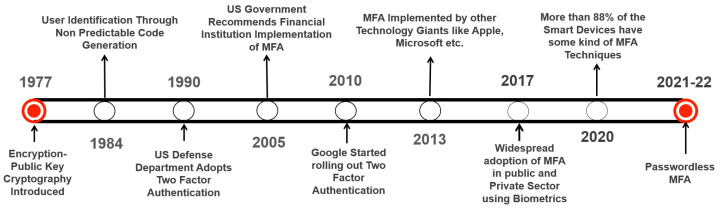
Key events in the evolution and adoption of MFA security mechanism.

In single-factor authentication (SFA), only one factor is used to validate the user. Owing to its simplicity and usability, SFA was readily adopted by users [57]. The earliest SFA mechanism is a password, which was used for the first time in the year 1961 [58]. However, as a single factor, a password is an inadequate security mechanism. Over time, SFA proved to be vulnerable to a number of security threats as authentication using only one factor did not provide sufficient security. Figure 7 presents the timeline showing the evolution of MFA technology [59,60].

Consequently, 2FA was introduced, which combines a knowledge factor (username and password) and user ownership factor (smartphone, computer) to provide an additional barrier for unauthorized users. 2FA implies that a user has to provide two credential proofs in order to gain access. The credit of the invention of 2FA sis contested by Kim Dotcom, claiming that he invented 2FA in 1997, and AT&T Company, which asserts that it invented 2FA in 1995. Regardless, AT&T was granted the patent before Kim. Though 2FA offers more protection than SFA, using a strong password is recommended.

Millions of people have suffered in recent years from stolen credentials from corporate databases. The relentless rise of cybercrime is so detrimental that by the year 2021, data breaches across the globe are expected to cost $6 trillion [58]. Moreover, a report by Consumer Sentinel Network Data Book 2019 reveals that from 2015 to 2019, identity theft attacks have increased more than three-fold. To offer a persistent and higher degree of security, multi-factor authentication (MFA) was introduced. Formerly, MFA systems were generally based on 2FA. MFA relies primarily on biometrics, a revolutionary authentication technique that identifies people through their biological characteristics. MFA offers quick, reliable, and user-friendly authentication. Recently, it has gained popularity in the cybersecurity sector. MFA was not adopted for quite a while by individuals and enterprises for reasons such as friction issues, high complexity, and cost. However, the omnipresence of smartphones accelerated the adoption of MFA. Smartphone features such as one-time passwords (OTP) via email or SMS, producing ownership (user’s phone) and biometric factors (face recognition, fingerprint scanning, etc.), as well as cloud technologies, contributed heavily to the extensive implementation of MFA. Moreover, the widespread use of 2FA by giant corporations such as Apple and Facebook led to global attention being paid to MFA. According to a report by Okta, 70% of corporations used two to four factors for security in the year 2018. Moreover, the study shows that 26% of people adopted MFA for personal use and 38% adopted it for professional use [61].

### 3.1. State of the Art in MFA

Several types of multi-factor authentication mechanisms and schemes have been proposed in recent years. The authors of [62] proposed a biometric-based multi-factor authentication system for securing online banking transactions on the cloud. Further, the authors proposed a mechanism for obscuring the personal and financial data of the users using anonymization and tokenization techniques. The authors of [63] proposed a two-factor authentication mechanism for securing online transactions. The proposed technique uses random codes generated via a mobile application in order to authenticate the legitimacy of the users. The authors of [64] proposed a three-factor authentication including a password, biometric, and tokens. The user is authenticated using the combination of all three factors. The authors of [65] proposed a new method to authenticate the users using network smart cards in prove the legitimacy for performing a secured online transaction. In [66], a new method to secure web accounts is proposed. The method works by generating a hash code using the shared number and current GPS location of the user. The scheme is a three-stage mechanism, in which the first stage requires the user to log in conventionally using the username and password. The second stage is the token-generation stage, wherein a hash-based token is generated using the pre-shared number and current GPS location of the user. The final stage is the authentication of the token, where the generated token in stage two is authenticated for legitimacy. The authors of [67] provided a novel multi-factor authentication mechanism using near field communication (NFC) based a smartphone, face (as biometric), and a four-digit pin. The whole process was divided into four modules. The first module performs the registration of the user with the system, it captures the face of the user, and the four-digit pin along with other personal details such as name and address. The second module is responsible for handling the transfer of user data to the receiver through NFC-based mobile phones. The third module is responsible for performing facial recognition of the user, and finally, the fourth module validates the pin entered by the user by matching it with the pin used at the time of registration. The authors of [68] discussed the security issues in electronic banking systems. They also provided the best practices that can be followed to avoid intentional and unintentional security threats and vulnerabilities. The study primarily focused on describing the interaction of the users with online systems. Further, it discusses the various security schemes such as SSL, TLS, and handshaking. The authors of [69] provided an X.509 v3 certificate-based improved approach for securing online transactions. The authors proposed using a smart card to store the certificate data, as well as a private key. Finally, the approach was theoretically compared with the classical SET approach. In [70], the authors proposed a GPS-based multi-factor authentication mechanism where the real-time GPS coordinates of the person are compared with the coordinates of the Point-of-Sale location apart from the login id and password authentication. The matching of GPS coordinates provides an extra layer of security to ensure that the user is legitimate and is the one performing the transaction. They also proposed identifying the distance between the user’s mobile device and the transaction device and if the distance exceeds the predefined threshold limit, the transaction is aborted. The authors in [71] proposed an improved SHA256 mechanism by integrating blockchain technology to secure the online transactions. They proposed creating smart contracts to ensure data integrity, non-repudiation, and trust among the participating entities of the online transaction. The authors in [72] proposed a novel protocol using transaction identification code and SMS to enforce an extra layer of security over the classical password-login system. In [73], the authors proposed an improved version of the authenticated key exchange (AKE) protocol by generating a dynamic ID to provide anonymity and enhanced security compared to the classical AKE protocol. The improvement makes use of two-factor authentication, which is computationally lighter in implementation and thus can be used in power-constrained devices. Furthermore, the authors compared the proposed approach with existing approaches in terms of functionalities and computational costs and found it to be effective in both. Table 2 provides a summary of these observations.

### 3.2. MFA Implementation Challenges

**Figure 8:**
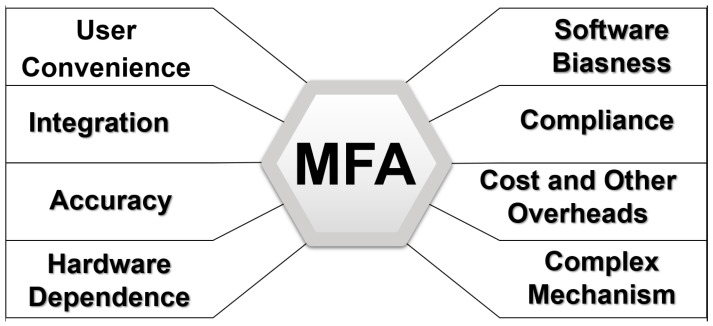
Issues and challenges in MFA implementation.

There are several internal and external challenges and issues involved with implementing MFA. Some of them are shown in Figure 8 below [57,79,80,81,82].

(1) *User Convenience*

User convenience is one of the most important factors that hinder the implementation of MFA. As the service providers are more inclined to provide a seamless user experience, the implementation of MFA can sometimes act as friction for the users, which makes them further wait to authenticate them. Therefore, MFA must not be implemented in all places but rather should be optimally implemented in appropriate services such as banking systems, online transactions, and confidential transactions.

(2) *Integration*

The integration of MFA mechanism in the existing legacy systems is a challenging task. This includes several issues such as system compatibility, speed, and scalability.

(3) *Accuracy*

The biometric-based MFA approaches are usually probabilistic in nature, and their accuracy tends to fluctuate with different inputs and conditions.

(4) *Hardware dependence*

Hardware-based MFAs such as fob keys, Biometrics, ECG, and EEG are prone to hardware failures, wear and tear, etc. Several other issues such as climate change and external environments also pose challenges in hardware-based MFA.

(5) *Software Biasness*

The program written for implementing the MFA mechanism may sometimes perform in a biased manner. For example, it may read faces and fingerprints of white people better than their Black counterparts. Therefore, software-based MFAs must be unbiased with respect to color, caste, or creed.

(6) *Compliance*

There are several internal, local, and international compliances associated with the implementation of MFA mechanisms. For example, some compliances restrict the implementation of MFA for specific services only.

(7) *Costs and other overheads*

The cost and other implementation overheads such as trained security professionals, incident response management, etc., further add to the implementation challenges.

(8) *Complex Mechanism*

The implementation mechanisms of MFA are highly complex and must be handled and managed by trained professionals only. This hinders its large-scale adoption in smaller businesses.

## 4. The Need for MFA in Smart Cities

The legacy credential-based mechanisms are no longer appropriate for the diversely connected IoT devices within smart cities. Since they are prone to hacking, there must be an extra layer of security to protect the entities in the smart cities. In the past, several notable security breaches have taken place, which prompt us to strive to provide MFA for better protection of smart city ecosystems.

### 4.1. A Few Notable Security Breaches in the Past

A lack of strong laws and regulations has already resulted in many security breaches over the years in history. In a bizarre incident, an IoT system in a casino was compromised through the temperature controller of an aquarium in that casino. The attackers encashed the vulnerability of the IoT device to gain access to the network and later retrieved the customer data through it. This incident was daunting, as nobody had thought of implementing security features in a simple temperature controller of an aquarium. In another major data breach, Equifax was hacked to gain information from over 143 million customers. The hackers took advantage of an Apache Struts vulnerability that was neglected by Equifax and was not fixed [83,84,85]. A large number of outdated and obsolete electronic devices and sensors are discarded as new IoT ecosystems are implemented. This generated e-waste has adverse effects on the environment. It is estimated that most consumers lack knowledge of the risks associated with the rich personal and private data stored in the connected devices, resulting in a low rate of resting and recycling of smart devices. This has a devastating effect on the environment. With such large-scale adoption of IoT into the mainstream, it has become an easy target for hackers and cybercriminals. Today, even a small loophole in the system can result in the shutting down of the entire IoT system, causing chaos and loss. As most facilities today are adopting this technology to their advantage, there is an increased risk of hacking and other similar security breaches. The past few years have seen many such attacks that were targeted at vulnerable IoT devices. The Mirai Botnet attack in the year 2016 took down the whole Internet [83,84,85]. The attackers took advantage of the outdated version of the Linux Kernel running on the devices. In a similar attack, named the “Brickbot attack”, the attackers targeted the connected devices that had not changed the default username and password settings [83,84,85]. Many such devices were targeted and eventually killed by the attacker. In November 2016, hackers successfully got into the heating controller system of two buildings in Lappeenranta, Finland, resulting in the failure of the heating system to raise the temperature of the entire buildings and causing the temperatures to fall below freezing point [83,84,85].

As the concept of the smart city is coming to reality, the everyday dependence on IoT devices and IT infrastructure is increasing to keep the automation updated. In a smart city setup, the city’s infrastructure relies on connected devices and services to control, monitor, and manage everyday processes such as traffic management, power supply, waste management, etc. The dependence on connected services makes the entire infrastructure vulnerable to cyber-attacks. The smart cities of today are dependent on automated computer-controlled systems that monitor and manage all services. In such a deeply connected setup cyber-attack will have a devastating impact. As per the reports of Lloyd’s city risk index, New York faced an economic loss of USD 2.34Bn from cyber-attacks in past years. In 2017, wannacry ransomware attack impacted thousands of devices across multiple cities using the Microsoft Windows operating system [86].

In March 2018, the city of Atlanta was hit by a ransomware attack [87]. The attack resulted in the shutdown of the WiFi at the Hartsfield Jackson airport. The impact of this attack was so strong that more than eight thousand employees were forced to shut their computers down. The ransomware attack directly affected the government and crippled the entire city of Atlanta for a few days.

In April 2019, the city of Stuart, Florida, suffered a similar fate when the Ryuk ransomware attack froze the city’s servers, which affected the payrolls, email, utilities, police, and other major services in the city [88].

In May 2019, the city of Baltimore was digitally seized by hackers using the RobinHood ransomware attack [87,89]. The hackers took control of over ten thousand government computer systems and demanded ransom in exchange. A similar attack was also faced by Greenville city in the same year [90]. In July 2019, Georgia’s court systems fell prey to a ransomware attack that impacted the computers in the administrative office of the court [91].

**Figure 9:**
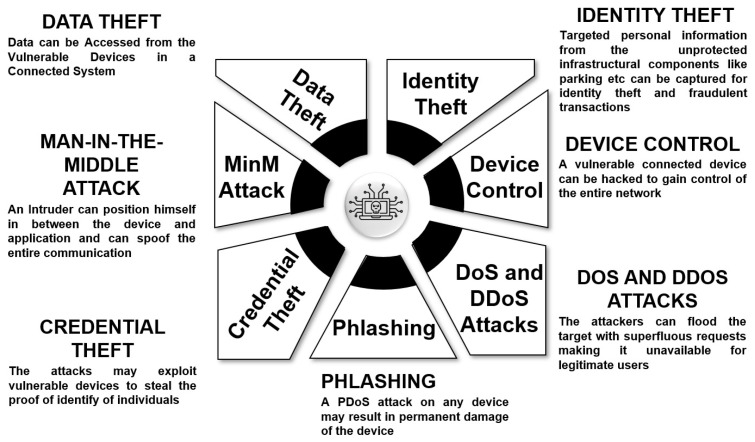
Types of attack on smart cities.

The dependency on technologies such as the Internet of Things, wireless sensor networks, and other connected technologies poses a potential threat to the security and privacy of smart city components. Some of the major types of attacks that can impact a typical smart city are shown in Figure 9.

### 4.2. Recent Measures Adopted in Curbing Security Loopholes

(1) *Regulation of IoT devices by the UK*

In view of the aforementioned security breaches, it is clear that there is an immediate need to frame laws and regulations that will govern the manufacturing of these smart devices and oversee the entire process of the implementation, monitoring, and management of IoT ecosystems and the underlying networks. The popularity of IoT and its deep penetration into everyday processes is a clear indication that this technology is here to stay. To overcome these security vulnerabilities, different countries are coming up with security mechanisms, regulations, and standards. Recently, the UK government has established new regulations for IoT devices. These are quoted below [92,93,94]:

- “All passwords for devices connected to the Internet must be unique and not resettable to factory settings”;
- “Device manufacturers must publicly provide contact details so that vulnerabilities can be reported and acted on ‘in a timely manner’”;
- “Device manufacturers must explicitly state minimum time frames for security updates either in store or online”.

The hardcoded passwords in typical IoT devices are static in nature and can be compromised with the help of any malware or virus attacks on the device(s). Moreover, several IoT device manufacturers generally use the same default passwords for all the manufactured devices, which are fairly simple and can be cracked easily with the help of brute force attacks. Once the hackers gain access to the devices, they can restore the devices to the factory settings and can easily steal or change the default password, making the device unusable. The provision in the proposed regulation to restrict the resetting of the devices to the factory defaults is a good attempt to protect against such hacking and brute force attacks. In addition to this, the password can be linked with any form of biometric authentication or can be made dynamic (instead of a typical static password). This can further enhance the security and make it harder for hackers to attack and compromise such devices. Furthermore, the security can also be enhanced at the root level by setting the default passwords as a combination of upper- and lowercase letters, numbers, and special characters instead of simple numbers (e.g., default password is set to “admin”, “1234” or “1111” or “0000”in most of the cases). For example, a unique default password can be anything such as “Ab1&” or “Bt3#” etc.

Apart from these measures, further possible regulations can be added as best practices to enhance the existing and proposed systems governing IoT ecosystems. It has been suggested that there must be several different networks within the premises of an IoT ecosystem, and no two critical devices can be on the same network. For example, door locks and electrical appliances must not be on the same network. The primary reason for this is to ensure that the devices that are more prone to wear and tear become easy targets for hackers because of their limited inherent security. Once these devices become defective, a third party is generally involved in the rectification and servicing of such devices, which poses several obvious security threats. Therefore, it is suggested that such susceptible devices be isolated from other critical devices and that different networks be created for these devices within the same IoT setup. Multiple networks will make it much more difficult for hackers and cybercriminals to hack into the devices or systems. It is also suggested that different networks be secured with different security mechanism and passwords. The regulations must be set up such that

- Once any smart device (IoT device) is defected, only authorized personnel must be contacted for the repair or rectification (if any).
- The defective IoT device must not be disposed of in the open, there must be a buy-back policy, and the manufacturing companies must ensure that any defective devices are taken back from the users and the replacement is delivered within due course of time to ensure seamless services and connectivity (just like electric meters in house-holds, for which users cannot just throw them away and buy new ones if they stop working).

Providing public contact details of vendors will be of limited help. Instead, a localized single point of contact must be identified and made accessible (through multiple means such as IVR, SMS, Call, emails, and physical means) to the general public where the security vulnerabilities, mishappenings, out-of-pattern devices behaviors, thefts, etc., can be reported and corrective actions can be taken in a timely manner.

Providing firmware security updates is essential because of the exponentially growing security threats and the emergence of new security vulnerabilities at both the hardware and the software level. Explicitly stating a minimum timeframe for security updates will help, but it can further be enhanced by providing a notification to the users for updating the IoT device firmware for available updates and security patches. It must be the responsibility of the IoT device manufacturers to provide such notifications throughout the lifetimes of the device(s). Furthermore, with every security update, it must be ensured that the default passwords (if any) also be updated and the notification for the same must also be sent to the users. Other possible best practices can be

- Using anonymous usernames and passwords and a transparent consent mechanism in which the users must be informed about the type and nature of the data collected by the devices, and informed consent must be taken in accordance with EU GDPR.
- Ensuring near-zero possibility of user or device profiling.
- Introducing insurance of information and data by the data processing enterprises.

Today, emerging technologies are boosting new business models, resulting in an increased demand to ensure the security and privacy of such technology-driven service models. With the rising demand for technological interventions in existing business models and processes, there is a need to regulate the laws around these technologies while ensuring a fair deal for all stakeholders and promoting innovation. The UK government has realized this need of the hour to regulate the most widely accepted technology of the last decade, the Internet of Things. This step by the government has sent a powerful message to the industry and other stakeholders that government intervention is likely in the cards in the near future. These laws have also ensured that the privacy and security of citizens will not be put at risk due to the irresponsible behavior of the manufacturers. The newly formed regulations will definitely help to remove the social barriers surrounding the adoption of consumer IoT devices and services, thus allowing larger sections of the population to benefit from the technology. Although it is too early to judge the success of these regulations, they can be analyzed from multiple viewpoints. On one hand, these laws are determined to create a safe ecosystem for IoT deployment, while on the other hand, they will also result in an added responsibility of the device manufacturers to comply with the guidelines and to incorporate the regulations into the already existing IoT ecosystem. In a nutshell, these laws have emerged as a ray of hope for a larger group of consumers and have paved the way for a safe and secure system that will ensure an increased adoption rate of technological interventions in the near future. However, there is still a need for comprehensive guidelines pertaining to these technologies that will ensure a safe and secure IoT environment without any added burden on device manufacturers and consumers.

(2) *State-of-the-Art Threat Monitoring Mechanism*

Monitoring the systems for any possible treats makes it possible for early identification of threats so that preventive measures can be adopted well in advance in the form of security patches and/or system updates. Using intrusion detection systems such as Albert can be deployed in the smart city network for identifying possible intrusion attacks and apply countermeasures [95,96].

(3) *Adopting Best Practices for Design, Implementation, and Maintenance*

A dynamically defined set of best practices can be adopted during the development of a smart city ecosystem [97,98]. All the stakeholders must be involved (taking inputs and feedback) in the development of such best practices, and they must sign off on the outcomes to ensure non-repudiation and fixing responsibilities.

(4) *Robust Incident Response Plan (RIRP)*

An incident response plan is highly important in critical systems such as those in smart cities. The queries that emerge include what needs to be done, who needs to be contacted, and how to proceed in the case of any mishappenings. All these questions are answered in an incident response plan. There are several points to be considered while drafting a comprehensive IRP [99,100].

  i. *Thorough Preparedness*

This involves identifying the goals, creating a well-trained pool of security professionals covering varied domains, assigning proper roles with respect to the skills and experience, creating holistic cybersecurity policies and ensuring proper implementation and monitoring, and ensuring proper information flow between the participating entities.

  ii. *Creating a Team of Experts and Quality Infrastructure*

Security professionals must be well qualified and experienced in identifying security loopholes and suggesting mitigation measures, and they must be equipped with state-of-the-art tools and techniques to counter any security breaches.

  iii. *System Monitoring and Detection of Breaches*

A good IRP must include comprehensive system monitoring tools that can identify any security breaches well in advance in order to take precautionary measures. The monitoring system must be able to identify different types of attacks, including phishing, DDoS, etc.

  iv. *Periodic Network System Check*

In order to ensure that the system is free of any internal or external attacks and is working well, it is imperative to periodically check the system’s health. Scheduling periodic system checks can mitigate security threats and associated risks.

  v. *Threat Removal Measures*

Once the threats are identified, a proper mechanism must be in place to remove them without affecting the normal system workflow.

(5) *Adopting Privacy by Design and Privacy by Default*

These two are the most preferred security features of modern-day IoT devices. The security features are built into the miniature IoT devices, which can take care of any security threats of a certain level and severity. This includes device anonymization, hard-coded security keys, and default passwords [101,102].

(6) *Mitigating Cross-domain Security Threats*

There are fair chances that a security threat may be overlooked or dodged at any level. Such security threats become more harmful and can affect the other units of the system. Therefore, a cross-domain security mechanism must be in place to protect the system from such unintentional security threats [103,104].

(7) *Protection from Distributed Denial of Service (DDoS) and Spam Attacks*
  i. - For Client-Server Architecture
    - CAPTCHA and reCAPTCHA

Distributed Denial of Service (DDoS) attacks are increasing day by day. As more IoT entities and cyber-physical systems are being compromised, they become resourceful for hackers to conduct massive DDoS attacks or spamming effects on servers to temporarily deny legitimate users from service. CAPTCHA is one of the earliest reverse Turing tests where a computer identifies the difference between a user and a computer or bot. It is carried out by giving a blurry or twisted set of alphanumeric characters that the user has to write back. However, people with low eyesight and vision feel confused while solving such CAPTCHAs. Moreover, the data used in CAPTCHAs are random and do not make any sense. reCAPTCHA is another similar technique provided by Google. However, this is often used for better purposes; for example, it uses old textbook abstracts and pictures for identification that make a dataset of a gold standard. Google reCAPTCHA v3 (score-based) and reCAPTCHA v2 (“I’m not a robot” checkbox) are the most used versions.

           b. Cloudflare DDoS protection

Since DDoS attacks involve multiple systems targeting a server to flood it with messages resulting in denial of service, enterprises have taken steps to mitigate the attack, and one such enterprise is Cloudflare, which provides DDoS protection by providing a reliable infrastructure, where it applies the DDoS protection on the Application Layer (Layer 7) of the TCP/IP model. It provides a network capacity of up to 42 Tbps, which makes it well-equipped to defend against any kind of substantial DDoS attack.

       ii. Peer-to-Peer (P2P) Architecture

Proof of Work (PoW) is a CPU-intensive algorithm that is run before submitting a transaction to the network [105]. It serves multiple purposes such as achieving consensus and mitigating DDoS attacks in the early generation of blockchain platforms such as Bitcoin and Ethereum. In this context, a computational puzzle is given, where the miner has to find the correct nonce value with a consecutive set of zeroes. A valid nonce is calculated by hashing transaction data with nonce values such that it leads to starting pre-defined consecutive zeroes. Miners are the ones to carry out this task in the case of the Bitcoin network and the Ethereum network 1.0. This algorithm is useful to prevent DDoS attacks, but it is not environmentally friendly. Moreover, PoW is slow and even requires some fees for miners to carry out such intensive algorithms on the user’s behalf. P2P networks ensure no single point of failure and better DDoS protection.

### 4.3. Role of MFA in Smart City Security

**Figure 10:**
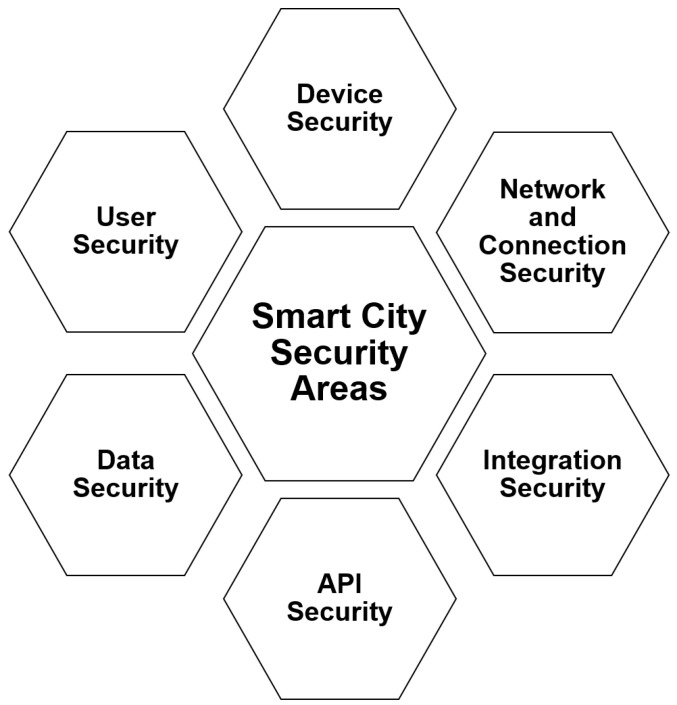
Smart city security landscape.

This section describes the need for MFA for smart city security. Here, we have discussed the role of MFA in providing an extra layer of security to every participating entity in the smart city ecosystem. Figure 10 shows the security areas with respect to the prime entities of a smart city [106,107,108,109,110,111,112,113,114,115,116,117,118,119,120,121,122,123,124].

#### 4.3.1. MFA for Device Security

Devices form the backbone of the smart city ecosystem. IoT-enabled devices that have embedded sensors collect information from the subject and their surroundings. The security of individual devices plays a vital role in the overall security landscape of smart cities. MFA can provide an extra layer of security to the devices to protect them from illegitimate access. However, how MFA can be included in power- and computation-constrained IoT devices is an area of research [106,107,108,109,110,111,112,113,114].

#### 4.3.2. MFA for User Security

Users are the most important entities of the smart city ecosystem, and thus the security of users is imperative. There are several MFA approaches that can be used to provide MFA to the users in order to protect their personal, financial, and other important details by authenticating the legitimacy of the users [106,107,108,109,110,111,112,113,114].

#### 4.3.3. MFA for Data Security

Data are another type of very critical entities of the smart city ecosystem. The amount of data at our disposal gives us the ability to achieve deeper insights into the systems, users, resources, and the services to provide extraordinary assistance to the end users. The more data there are, the greater the ability to extract novel insights to provide more engaging services and smarter decisions, along with identifying efficient ways to complete tasks. Thus, securing the data captured by multiple sensors spread across the smart city is a very important and intricate task. To ensure the security of such captured data, several innovative mechanisms have been devised in recent years, as discussed in [106,107,108,109,110,111,112,113,114].

#### 4.3.4. MFA for API Security

APIs play an important role in smart systems. With the help of APIs, we can directly access the data generated by sensors. Therefore, it is imperative to secure the API from any illegitimate access. Since most of the APIs follow REST or SOAP-based implementation, the corresponding security features may be adopted during its implementation [115,116,117,118,119,120].

(1) Tokens

Hardware and software tokens can be provided to establish trusted identities and provide role-based access to services and resources by using these tokens.

(2) Encryption and Digital Signatures.

Encryption and digital signature provide security of the data and ensure that only legitimate users are decoding, accessing, and modifying the data whenever needed.

(3) Vulnerability Identification

Regularly updating the systems to ensure any known and unknown vulnerabilities are identified and handled properly. Providing periodic security patches helps in strengthening the system against every increasingly novel security threat. Sniffers can also be used to identify vulnerabilities and data leaks in the system and network.

(4) Restricting API usage

As a precautionary measure, smart city designers can restrict the frequent calling of the API while keeping a check on its historical usage as well. Throttling can be used for protection against DoS attacks.

(5) Installing API Gateways

An API gateway provides the ability to authenticate the traffic on the API as well as ensure optimal usage of APIs.

(6) Using Wireless Application Firewall (WAF)

These are the most preferred means to protect the API’s platform. They facilitate secure communication between applications using a set of protocols for HTTPS.

(7) Using SSL/TLS

SSL and TLS can be used to protect the API from commonly known injection attacks on the APIs.

#### 4.3.5. MFA for Network and Connection Security

The functions of smart cities are completely dependent on the underlying network connectivity. A minor glitch in the network may cause severe damage to the smart city ecosystem at times, compromising the security of the users and data in transition. Therefore, it is imperative to secure the network and connectivity channel through encryption and other necessary security measures. In order to do so, MFA must be implemented to ensure the authenticity of the entities before the start of the communication, in between the communication, and just before the termination of the communication [115,116,117,118,119,120].

#### 4.3.6. MFA for Integration Security

Within a smart city ecosystem, decisions are dependent on the data coming from multiple sources. Several times, the decisions are the result of the integration of the data captured by multiple sensors. Performing the analysis and deriving a decision from it requires secure integration of the data from multiple sources. The integrity, authenticity, and veracity of the data must be maintained at any cost in order to provide a legitimate decision [115,116,117,118,119,120].

## 5. BAuth-ZKP: A Smart Contract-Based MFA (Proposed Approach)

BAuth ZKP is a mechanism that attempts to enhance the capabilities of the classical username-and-password authentication approach. The proposed mechanism uses the concept of ZKP and smart contracts for storing the authentication credentials. Here, instead of storing the actual password, the count of each character of the password is stored along with the Ethereum Address of the user. This character count is then hashed and sent to the blockchain storage. The reason for using blockchain-based smart contacts is to ensure a secure, transparent, and tamper-proof authentication. The proof of concept of the login process and authentication mechanism has been developed to showcase the working effectiveness. The unique features of blockchain technology makes it an appropriate solution for curbing the security loopholes in smart city ecosystems. With features such as transparency, immutability, P2P, and distributed architecture, blockchain technology can provide unprecedented security features with much improved Quality of Service (QoS) [125,126,127,128,129,130,131,132,133,134,135]. The consensus mechanism of blockchain provides a mutually agreeable set of instructions and rules for collaboration and interaction in a secure and non-repudiation manner. Blockchain can be used in the supply chain and the tracking of goods and services, providing faster, secured, and legitimate interaction among the participating entities [125,126,127,128,129,130,131,132,133,134,135,136,137].

Smart contracts can permanently replace the traditional database dependency that remains solely responsible for storing user sign-up/sign-in credentials. These credentials are vulnerable to many outsider as well as insider attacks. Often, these critical user credentials are used by employees for mimicking users for post-deployment testing and training purposes. The method of testing in this way reveals PII about the user such as name, home address, phone number, email-id, spouse details, etc., that expose the user to a wide range of risks. Insider attacks and personal attacks are key issues in traditional kinds of systems. Enterprises have invested a considerable amount of money and time in solutions for authentication. With each new epoch or layer of authentication, new vectors of data are collected from the user, which overlay more security and privacy concern. Any leakage of such critical data will have a detrimental impact on the user.

BAuth, which stands for Blockchain Authentication, is similar to OAuth (Open Standard for Access Protocol). This system uses the capabilities of Ethereum Blockchain and its Turing-complete program called Smart Contract. There are two crucial functions for authentication, namely Registration (Sign-Up) and Login (Sign-In), as depicted in Algorithms 1 and 2.
**Algorithm 1: Algorithm for Sign-Up/Registration Process****Input:** Ethereum Address and Password **Initialization:**
a)Initialize Metamask wallet account with faucet Etherb)Connect to Ropsten Test Network
**Function register**(*user_password*):account = web3.currentProvider.selectedAddresspromise = addAuth(user_password).send(from: account)**if** (*promise*) **then**print(“User Registered”)result = true**else**result = false**return** result**Function** submitfunction(*userpassword*):**if**(userpassword) **then**c = character_count(userpassword)registered = register(userpassword)**if** registered **then**store c in database mapped with Ethereum Address**else**print (“Enter Password”)


**Algorithm 2: Algorithm for Sign-In/Login Process**
**Input:** Ethereum Address and Password **Initialization:**a)Initialize Metamask wallet account with faucet Etherb)Connect to Ropsten Test Network
**Function** login(*user_password*)account = web3.currentProvider.selectedAddressuser_exist = check(account).call()**if**(*user_exist*) **then**result = loginAuth(user_password)**if** (result == true) **then**//user is authenticated//redirecting to Rest API server calls for ZKP verificationevaluate c’ = character_count(user_password)and send c’ to server for ZKP verification**if** (c == c’) **then**print(“User is verified successfully”)**else**print (“User is not verified”)**else**print (“Password do not match”)**else**print(“User not registered”)**Function** submitfunction(*userpassword*):**if** (*userpassword*) thenlogin(userpassword)**else**print (“Enter Password”)

**Figure 11:**
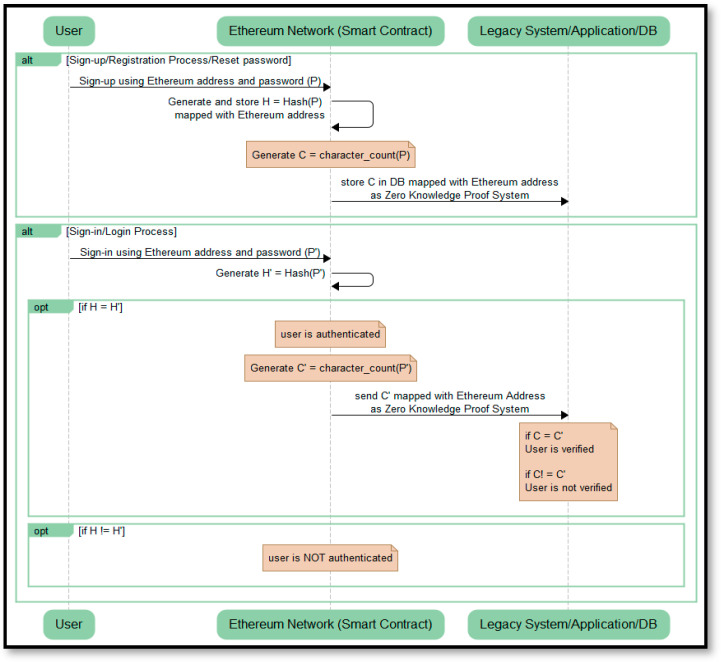
Sequence Diagram for BAuth-ZKP.

The above two algorithms are based on ZKP-based smart contracts. We have used this system as a process of a commitment scheme, which is an integral part of any ZKP mechanism. Hence, only the user authenticated by BAuth MFA is allowed to prove their verifiability to the server using the character-count zero-knowledge proof mechanism already in place. The sequence diagram of the flow of communication of the proposed BAuth-ZKP approach is shown in Figure 11.

Here, the users need to sign-up to the system for the first time and subsequently can sign-in to access the services. The sign-up requires the user to use their Ethereum Address in combination with the chosen password. The password (P) provided by the user at the time of registration (sign-up) is then hashed and analyzed to calculate the character count. This calculated character count is then mapped with the Ethereum Address and stored in the blockchain database as a part of ZKP system. At the time of login, this hashed password is matched with the entered password, and if found to be the same, the character count of the entered password is calculated and matched with the character count of the password stored at the time of sign-up. Once they both match, the user is verified and authenticated; else, the user is not verified.

We aim to present a new privacy-preserving authentication method, the “BAuth-ZKP: Smart Contract Based Authentication” solution. The process of sign-up and sign-in is simple, fast, and highly secure. Only the valid user has control over their credentials, which are signed by their Metamask Wallet, a browser extension-based wallet. Algorithms 1 and 2 are the algorithms for the same developed techniques.

**Figure 12:**
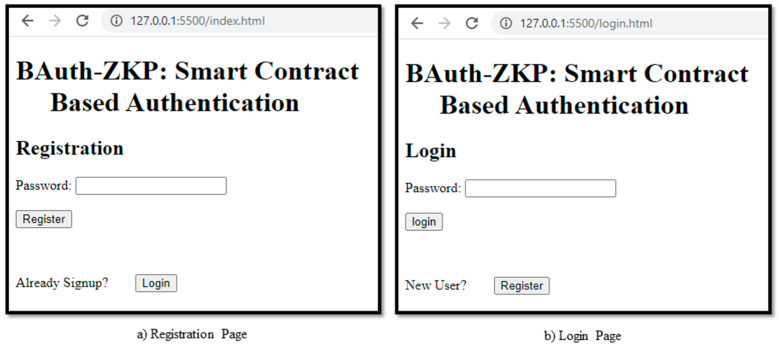
Proof of concept registration and login webpages of BAuth-ZKP.

**Figure 13:**
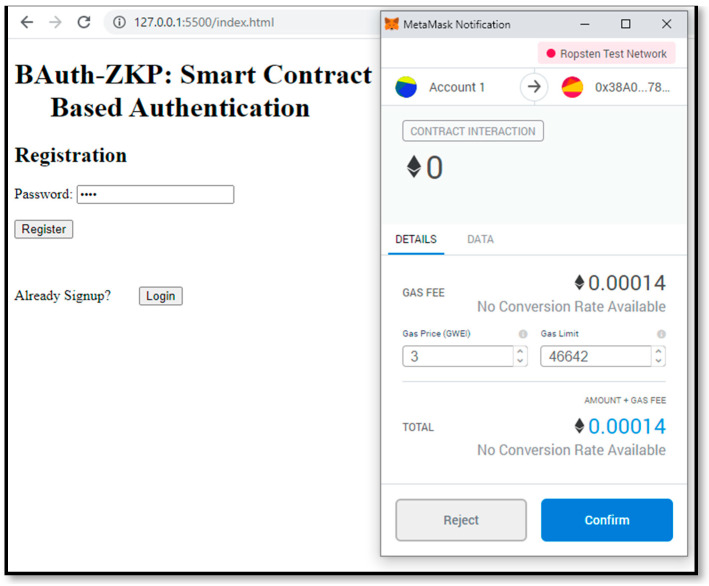
Confirmation window during the registration transaction using Metamask Wallet.

**Figure 14:**
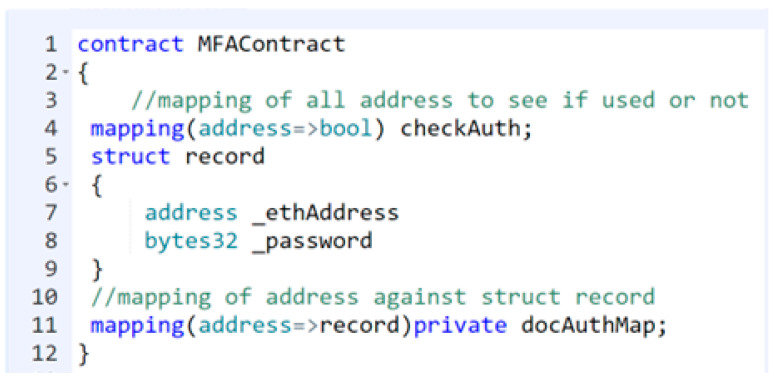
Code snippet of BAuth-ZKP MFA Smart Contract.

These algorithms are highly efficient and yield better results than traditional technologies. The proof-of-concept authentication application for the BAuth-ZKP mechanism is shown in Figure 12a,b. In cases of registration, the user can type in their password, and upon clicking the “Register” button, a popup Metamask window will open to confirm the transaction, as shown in Figure 13. In the proposed BAuth-ZKP approach, the user’s actual password is no longer stored on the cloud and only the character count set of the password is stored. Hence, even if servers are attacked or hacked, there is nothing to steal from. This can act similarly to OAuth; i.e., the open standard for authentication. Smart contracts are automated contracts that run on the Ethereum Network [138,139]. They are written in Solidity programming language, and the code snippet of our BAuth-ZKP MFA smart contract is shown in Figure 14.

**Figure 15:**
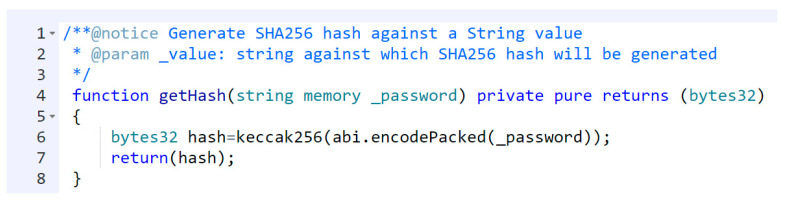
Code snippet of Hashing.

We used the SHA256/Keccak256 hashing algorithm for hashing the user password provided into the smart contract function getHash(), as shown in Figure 15. Hence, this way, the user’s original password is not shared publicly, and only the hash is stored on the blockchain.

**Figure 16:**
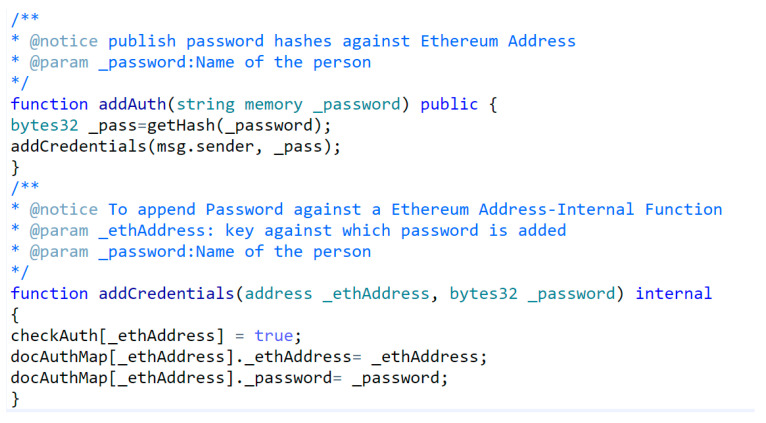
Code snippet of Adding Auth Credentials.

Here, users can add/store the hash of their password into the MFA smart contract using Metamask, as shown in Figure 16. The password will be stored against the key, which is the user’s Ethereum Address. All users have to maintain their Metamask wallet safely and refrain from sharing their password with anyone. In addition, this account should not be used for any crypto trading or exchange, since the anonymity is not ensured in this. Any enterprise or company can easily map the Ethereum address with the real identity of users. Hence, no crypto exchange should happen from the account used for interacting with the MFA smart contract, as it could lead to multiple types of attacks.

**Figure 17:**
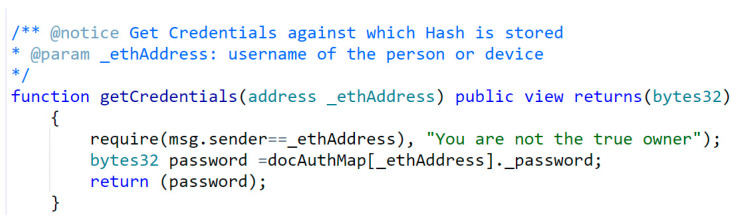
Authentication process Code Snippet.

In Figure 17, only a valid user can ask for its password hash from the MFA smart contract using the function getCredentials(). Here, only the authenticated user can request their password, and no one else can read it.

In the proposed BAuth-ZKP approach, blockchain technology is used such that only the authenticated users can request the hash of the passwords stored on the blockchain and the intruders if any are not able to access it, hence providing authentication security to the users, services, and devices. Since the sign-up and sign-in functionalities are based on smart contracts of the blockchain, only legitimate users are allowed to perform these operations.

### 5.1. Observations about and Analysis of BAuth-ZKP

(i) The same account (Ethereum address) as the Metamask should not be used to interact with any other cryptocurrency project, as our solution does not currently offer user anonymity. This is due to the fact that the external KYC (Know Your Customer) is applied and mapped with the user’s Ethereum address. Hence, it is the responsibility of the user to never use same account anywhere else.

(ii) Reset password and forgot password are easily manageable as users can re-register themselves by going to the registration page to submit the new password. A new transaction will be initiated, for which the user has to pay the gas fees as it is a transaction (changing the state of the blockchain), and the new password hash will override the previous password hash.

(iii) Once a user is registered into the platform, they are not required to pay any fees while logging into the system. The login process is absolutely free and instant.

(iv) Gas price and Transaction Type Details

We have analyzed the economic factor of our BAuth Smart Contract solution, as listed in Table 3.

We can use any Ethereum Provider wallet, but the most commonly used is Metamask as it is easy to use with the Web3 library while building applications. There are some other types of wallets, and each provides an advanced mechanism of MFA nowadays, as discussed below in Table 4 [140].

Blockchain uses wallets to check the cryptocurrency balance and make transactions. These wallets are software packages that allow transactions to be made on the blockchain. For any transactions in the blockchain, the users need to use the wallet by unblocking the funds in the wallet. This can be performed by using the keys. Wallets do not store the currency; rather, they help in interactions with the blockchain. The coins move from one address to another and never leave the blockchain. Different wallet types provide some additional features such as user authorization, managing and generating keys, crypto exchange, and user anonymity. Crypto wallets can be broadly classified as software wallets and hardware wallets. Software wallets come as software programs for the PC or mobile phones, while hardware wallets come as physical vaults for storing the data on hard drives. Based on the internet connectivity, these wallets can be further classified as hot and cold wallets. Hot wallets are connected to the internet, while cold wallets are offline. Since nothing is fully safe and secure on the internet, hot wallets are at constant risk of theft. These crypto wallets store currency-specific public addresses to facilitate transactions. This helps the clients to read and submit the transactions on the blockchain. A private key is associated with all public addresses that allow the wallet to be accessed.

With many different wallets, it is important to choose the appropriate wallet to cater to the user needs. A universal cryptocurrency wallet that supports multiple cryptocurrencies is always a better choice. Typical crypto wallets include the following [140].

Software wallets: These are digital wallets that can be downloaded and used on PCs and mobile phones. These types of wallets are convenient but are prone to theft and security issues.

Hardware wallets: These are physical devices that are used to store cryptocurrencies. These wallets are secure and less vulnerable, as the private keys are stored on the hardware device, which is not connected to the internet.

Online wallets: These are software programs that run on the cloud. These can be accessed from any device with an internet connection, but they are less secure because the private keys are stored on a third-party server.

Multi-signature wallets: These wallets require multiple private keys to access the funds, which adds an extra layer of security. However, they require coordinating with other people to access funds.

### 5.2. Other Blockchain-Based MFA Approaches

#### 5.2.1. Hydro Raindrop MFA Platform

Hydrogen or the Hydro Raindrop is a 2FA solution built to provide layered authentication and security [141]. It can be implemented in multiple ways with the application as well as with WordPress websites. This application has self-explanatory documentation that explains its advantage over the Google Authenticator Application. The proposed BAuth-ZKP solution, however, is easy to use and integrate and is built on top of Ethereum Blockchain. With more advancement in the Blockchain ecosystem, more enhanced MFA techniques are bound to follow.

#### 5.2.2. IOTA Access

Technology advancement is a wave traveling faster than ever. Multi-factor authentication is further supported by new means of access and authentication methods. IOTA Access is another revolutionary project provided by the IOTA Foundation [142]. It enables access control over devices to users only when certain conditions are triggered. It is a lightweight framework offering its solution to embedded devices, as well as to any smart infrastructure in a smart city. It is secure, and no transaction fees are paid, as it uses Tangle protocol. It was launched in September 2020 and is not yet production-ready. IOTA Access is divided into four further repositories, namely access SDK, access server, access mobile client, and access policy store. IOTA Access is a followed-up project to XAIN’s Frost project. IOTA Access proclaims to be the newest innovative way of multi-factor authentication through the distributed ledger and tangle altogether in a secured and reliable way.

## 6. Conclusions, Discussion, and the Way Ahead

In modern smart city ecosystems, where rich personal and financial information and other data are being digitally shared amongst the government, businesses, and residents, security is paramount to create trust amongst the stakeholders. Furthermore, the unprecedented effects of COVID-19 on all sectors have witnessed a sudden surge in online transactions. People around the globe are preferring online modes for doing transactions. From doing business to managing the financial and banking systems and managing the critical nuclear, hydro, and electric systems, the digitization of processes has created an urgent need to enhance security against cyber-attacks and data thefts. The increasing dependence on internet technologies has led to new issues and security challenges. With the growing innovations and developments in the domain of technology, cyberspace has become more vulnerable than ever before. The use of rudimentary password-based approaches to secure an account has become ineffective and inefficient in addressing increasing cyber-attacks and cybercrimes. The use of a single password for multiple accounts is very common amongst users, which makes it vulnerable as it may lead to a series of attacks on several accounts of the users. Furthermore, with state-of-the-art systems and other resources at their disposal, cybercriminals and hackers are coming up with innovative ways to take advantage of the vulnerabilities of the classical security approaches to commit crimes. In recent years, multi-factor authentication mechanism has come as a ray of hope for safeguarding against cybercrimes. It is based on the concept of using more than one credential for authentication and can help to ensure security against cyber-attacks and crimes. Advanced MFA mechanisms have the potential to provide a seamless user experience while ensuring the security of the online transactions by providing an extra layer of security. One of the most popular MFA systems is two-factor authentication (2FA), where a user needs a password and a one-time-generated security token such as OTP to access the account. These types of authentication systems are popular in banking and online transactions where a one-time password is required along with login credentials. However, many users find it to be an overhead to perform one extra step in the verification process. Furthermore, with hackers finding new ways to obtain illegitimate access to users’ accounts, two-factor authentication is also vulnerable to interception and replication attacks.

This paper discusses the various aspects of using MFA in a smart city ecosystem. Further, a new smart-contract-based authentication solution named “BAuth-ZKP” is proposed and presented that uses an authentication protocol where, instead of the actual password, only the character count set of the password is stored on the server, which ensures the privacy and security of the PII of users in cases of server attacks and hacking. The proposed method not only protects the passwords from being hacked but also makes them obscure to intruders (if any). It uses the concept of Zero-Knowledge proof along with blockchain-based smart contracts for performing authentication of the users.

**Figure 18:**
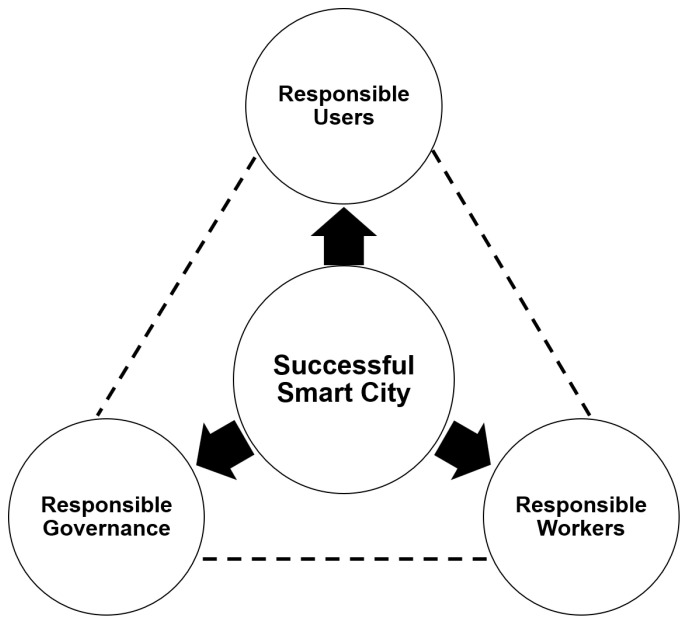
The golden triangle for the success of a smart city.

Security experts and developers are trying hard to provide a seamless experience to users while ensuring the state-of-the-art security to them. Recently biometric-based passwords have gained significant popularity amongst the masses. With smartphones and other devices coming up with biometric security such as fingerprint, facial, voice, and iris recognition integrated into systems, the traditional password-based approaches and 2FA methods are slowly fading away. The secret double octopus provides a modern MFA that eliminates the storing of any secret such as a password. This type of authentication is performed over multiple channels using a different security method on each channel. In the current scenario, where the smart city concept has gained significant popularity, there is a need to implement effective and trustworthy security mechanisms for successful digitization. The success of the smart city ecosystem is based on three prime factors, including: “responsible users”, “responsible governance”, and “responsible workers”, as shown in Figure 18.

The synchronization between these components in a transparent, trusted, and privacy-preserving manner can pave way for the realization of a smart city in the true sense. With the increasing dependency on the Internet and related technologies, the whole world is transitioning towards a digital era. This paradigm shift requires enhanced authentication and authorization mechanisms to keep a check on increasing cybercrimes and data theft. Today, existing MFA mechanisms such as OTPs are combined with biometric-based authentication to provide an added layer of security to the users. Behavior-based Authentication (BAuth), developed by neXenio [143], is a kind of mobile app that can be used to sense individuals’ gait movements, and if there is any change in the gait pattern, a notification can be triggered, alerting the user of any security threats. BAuth monitors the usage pattern of the user and assigns a confidence score to different activities. This is to identify whether the present user of the device is the actual owner or not. If the confidence score does not match, the user is alerted. As per the reports in [144], the users have become more responsible and aware of the security threats around them. However, since a smart city is a networked ecosystem, even a single weak entity can cause devastating damage to the whole system. Better measures must be adopted at all levels, including the government level (policies and regulations), the organization level (incident response, best practices, and access levels), and the individual level (self-alertness, resilience to adopt new features, and work ethics) to secure each individual entity of the system; only then can a secure and privacy-preserved smart city be sustained. These measures must be feasible and frictionless and should not add to the overhead for users and organizations. Extensive research is going on in the field of MFA, and in the future, behavioral patterns of individuals such as gait, gestures, body language, and voice tone can be used to provide MFA-based enhanced security. Furthermore, MFCC-based tonal quality analysis can be performed to provide voice-based MFA. Modern MFA techniques must provide a unified and frictionless experience to users and at the same time must be affordable and easily integrated into the legacy systems. Currently, most MFA approaches are device-centric, where we tend to authenticate the device rather than the user. In the future, MFA must be focused on authenticating the identity of the users rather than the devices. As a future scope, mechanisms need to be explored that can be used to provide multiple synchronous ways of authenticating the users of the system. This may include technologies such as behavioral biometrics and natural-language-processing-based pattern recognition, which are more user-centric. However, these mechanisms have their own limitations, including setup costs, user dependency, and weather conditions.

## Figures and Tables

**Table 1:** Summary of Important Discussions on Smart City Security.

S.No	Reference	Security Areas	Key Discussions
1	[30]	Cybersecurity attacks and their impact on smart cities.	Presents a secure IoT-based architecture for smart cities. Additionally discusses the cloud-based architecture for smart cities.
2	[33]	Detecting anomalies, threats, or unwanted behavior. Improving the current systems. Predictive policing	Discusses three categories of security interventions in smart cities that combine new sensors with traditional actuators, seeking to make legacy systems intelligent and introducing novel metrics and notions of mass information and crowd-sourcing along with threat prediction.
3	[34]	Data privacy, confidentiality, and integrity. Authentication, authorization, and accounting. Single points of failure. Denial-of-service attacks.	Discusses the security issues in IoT layered Architecture, categorized into low, intermediate, and high levels.
4	[35]	IoT design challenges such as availability, reliability, interoperability, scalability, and performance.	Presents an IoT architecture model in the context of smart city applications.
5	[36]	Smart city challenges such as design and maintenance cost, heterogeneity, and data privacy.	Overview of smart cities along with the key features and characteristics, generic architecture, composition, and real-world implementation aspects.
6	[37]	Challenges of IoT-based smart cities such as security and privacy, legal and social aspects, and demand response barriers.	Discusses the concept of smart cities and the applicability of the Internet of Things.
7	[38]	Security, privacy, information security, infrastructure challenges, and management and processing of personal data.	Develops a smart city interaction framework and discusses the concept of smart cities in view of privacy and security, infrastructure, energy, healthcare, operational threats, and use of blockchain technology.
8	[39]	Privacy and security issues. Diminishing freedom of speech and democracy.	Provides systematic insight into and understanding of the negative and positive results of the development of smart cities.
9	[40]	Safety and security in smart cities using AI,	Discusses AI-based smart solutions for smart cities.
10	[41]	Biometrics. Digital image forensics. Digital audio forensics. Deep fakes.	Provides a review of the digital forensic schemes for audio–visual biometric data used by the applications designed for smart cities.
11	[42]	Cyber threats within and outside an organization. Sophisticated, coordinated, and targeted cyberattacks including advanced persistent threats.	Focuses on the CPS in smart cities and discusses the concept of cyber resilience and DFIR aspects with the help of a systematic literature review.
12	[43]	Privacy aspects of smart cities.	Discusses the state of the smart cities around the globe and focuses on the privacy aspects, focusing on individual privacy and potential solutions and privacy-enhancing technologies.
13	[44]	Authentication and authorization	Discusses the various IoT authentication methods in a smart city system and highlights the concept of multi-factor authentication.
14	[45]	Privacy risk classification based on location, state of body and mind, behavior and action, social life, and media.	Focuses on the privacy-enhancing technologies in a smart city system. Additionally presents the already existing smart city applications with possible privacy issues and potential solutions
15	[46]	Security and privacy aspects of the collaboration between drones and IoT.	Discusses the role of drones and IoT in smart city applications such as smart communication, smart transportation, smart agriculture, disaster management energy saving, and e-waste management.
16	[47]	Monitoring applications in a smart city system.	Focuses on the system design, configuration, and running in a smart city.
17	[48]	Issues and challenges in the integration of blockchain technology in smart cities.	Discusses the potential of using blockchain technology in the improvement and development of a smart city.
18	[49]	Issues related to data dissemination in a smart city	Presents a data-centric view highlighting the various data management techniques for consistency, interoperability, granularity, and reusability of the data collected from the IoT devices. Additionally highlights the machine learning and deep learning techniques in smart cities.
19	[50]	Security threats in a smart grid.	Discusses the security aspects of smart homes and smart grids in a smart city system and highlights the countermeasures for the security threats.
20	[51]	Unanticipated security threats, data manipulation attacks, DDoS attacks, privacy issues.	Discusses the various aspects of the deployment of cyber-physical systems in smart cities such as security, privacy, technical issues, policies, and governance.
21	[52]	Privacy issues related to botnets, virtual reality, AI, and driverless cars. The security aspects related to authentication, confidentiality, integrity, etc.	Discusses the privacy and security issues in the currently existing smart city systems and highlights the requirements to develop a secure smart city
22	[53]	Cyber security challenges for smart cities. Potential threats in various smart city sectors such as transportation, government, and healthcare.	Presents a review of the recent smart city projects and initiatives around the world and discusses the vital design notions of a smart city.

**Table 2:** Observation and Analysis of Related Research Works on MFA.

S.No	Reference	Approach	Key Features	Observations
1	[74]	NetCents	Vendor-independent floating scripts for performing transactions, specifically micropayments	NetCent works in offline mode
2	[75]	MFA for cloud computing	Multi-factor authentication using arithmetic CAPTCHA, Cloud services, and resources are divided into 3 categories	The selection of IMEI number as a secret key is not appropriate as IMEI is a fixed number that can be accessed in case the mobile is stolen.
3	[57]	Survey of MFA approaches	Several types of MFA approaches have been surveyed, including biometrics, facial recognition, iris recognition, etc.	The future of MFA Integration includes factors such as behavior, thermal imaging, EEG analysis, BFT, OCS, and DNA analysis. These approaches were compared for 6 aspects and performance parameters
4	[76]	Dynamic selection of multi-factor authentication factors on the basis of scenario and requirements	To develop a dynamic MFA strategy, previous authentication factors were observed on the basis of performance, limitations, and other constraints	Trustworthiness of different authentication factors was analyzed for the development of a dynamic MFA strategy
5	[77]	CNN-based Anti-Spoofing MFA	A novel hybrid technique is proposed for user authentication, along with identifying whether the user has spoofed the system during authentication or not. A CNN-based model is used to identify the spoofing of biometric data.	The model was tested on 5 benchmark datasets and the results show promising results
6	[78]	MFA for preventing impersonation attacks in MANET	The vulnerability of ithe mplicit linking of the actual node device with the actual entity in MANET is identified for possible impersonation attack, and an MFA framework is proposed to overcome this shortcoming using certified keys and certified node characteristics.	Extra sensing capacity is required for gaining further improvement in authentication. Thus, it consumes more power.

**Table 3:** Smart Contract transaction types and gas details.

Function Name	Function Type	Avg. Gas Used in Eth	Avg. Price in (USD) *	Avg. Price in (INR) *
Contract Deployment	Transaction	0.000432	0.54	46.65
addAuth	Transaction	0.000127	0.16	13.71
Login	Call	Nil	Nil	Nil

* (prices as of 9 January 2023. Source: Coinbase).

**Table 4:** Types of Wallets.

Type of Wallet	Name of Service
Desktop Wallet	Mist, Ethereum Wallet, Exodus, Gnosis Safe Desktop Wallet
Web-Based Browser and Mobile Wallet	Metamask, MyEtherWallet, Gnosis Safe Web & Mobile Wallet, Monolith Mobile Wallet, Argent Wallet, Squarelink, Torus, Portis Mobile Wallet, Rainbow Mobile Wallet, Crypto Exchanges Wallet (Binance, COSS, Coinbase), Dharma Wallet, MyCrypto
Hardware wallet	Trezor (Trezor One & Trezor Model T), Ledger (Ledger Nano X & Ledger Nano S), Wallet.io

## Data Availability

Not applicable.
